# Supplementary material for: Advances in analytical approaches for background parenchymal enhancement in predicting breast tumor response to neoadjuvant chemotherapy: A systematic review
Source: PLoS One. 2025 Mar 7;20(3):e0317240. doi: 10.1371/journal.pone.0317240 (PMC11888135; doi:10.1371/journal.pone.0317240)
Supplement: S2 File — Search strategy for the systematic review of advances in analytical approaches for background parenchymal enhancement in predicting breast tumor response to neoadjuvant chemotherapy. (DOCX) [file pone.0317240.s002.docx]

**S2: Search strategy for the systematic review of advances in analytical approaches for background parenchymal enhancement in predicting breast tumor response to neoadjuvant chemotherapy**

| PICO | Concepts | Related terms for search |
| --- | --- | --- |
| Population/  Problem | Patients with breast cancer whose background parenchymal enhancement was measured at different time points during neoadjuvant chemotherapy  Inaccurate or untimely prediction of breast tumor response to NAC | (Background parenchymal enhancement OR parenchymal enhancement OR BPE) AND  (Breast tumor OR breast cancer) AND (neoadjuvant chemotherapy OR NAC) |
| Intervention | Analytical approaches or methods for assessing BPE changes based on dynamic contrast-enhanced MRI | (BPE analysis) OR (Background parenchymal enhancement changes OR BPE changes) OR (Dynamic contrast-enhanced MRI OR DCE-MRI) |
| Comparison | N/A | N/A |
| Outcome | Studies related to BPE analytical approaches, advancement in BPE analysis, limitations in the current analytical methodologies, and strategies to address identified limitations. | (Background parenchymal enhancement analysis) OR (BPE analysis) |

*Note:* The comparison was not included in the search terms because no analogous investigations were identified to serve as a benchmark.
