# Supplementary material for: Advances in analytical approaches for background parenchymal enhancement in predicting breast tumor response to neoadjuvant chemotherapy: A systematic review
Source: PLoS One. 2025 Mar 7;20(3):e0317240. doi: 10.1371/journal.pone.0317240 (PMC11888135; doi:10.1371/journal.pone.0317240)
Supplement: S3 File — Search Terms as per Databases during Study Search. (DOCX) [file pone.0317240.s003.docx]

**S2: Search Terms as per Databases during Study Search**

| Database | Search terms | Articles retrieved |
| --- | --- | --- |
| PubMed | (Breast tumor OR Breast cancer) AND (background parenchymal enhancement OR parenchymal enhancement OR BPE) AND (neoadjuvant chemotherapy OR NAC) OR (BPE analysis OR background parenchymal enhancement changes OR BPE changes OR ‘BPE assess) OR (Dynamic contrast-enhanced MRI OR DCE-MRI) AND (predict*) AND (response) | 760 |
| Cochrane Database of Systematic Reviews (CDSR) | (Breast tumor OR breast cancer) AND (background parenchymal enhancement OR parenchymal enhancement) AND (neoadjuvant chemotherapy OR NAC) | 1 |
| Google Scholar | (Breast tumor OR Breast cancer) AND (background parenchymal enhancement OR parenchymal enhancement OR BPE) AND (neoadjuvant chemotherapy OR NAC) OR (BPE analysis OR background parenchymal enhancement changes OR BPE changes OR ‘BPE assess) OR (Dynamic contrast-enhanced MRI OR DCE-MRI) AND (predict*) AND (response) | 110 |
| IEEE Xplore | ("Breast tumor" OR "breast cancer") AND ("background parenchymal enhancement" OR "parenchymal enhancement" OR "BPE") AND ("neoadjuvant chemotherapy" OR "NAC") OR ("BPE analysis" OR "background parenchymal enhancement changes" OR "BPE changes" OR "BPE assess") OR ("Dynamic contrast-enhanced MRI" OR "DCE-MRI") AND (predict*) AND (response) | 11 |
| **TOTAL** |  | **882** |
