# Supplementary material for: Advances in analytical approaches for background parenchymal enhancement in predicting breast tumor response to neoadjuvant chemotherapy: A systematic review
Source: PLoS One. 2025 Mar 7;20(3):e0317240. doi: 10.1371/journal.pone.0317240 (PMC11888135; doi:10.1371/journal.pone.0317240)
Supplement: S4 File — Data extraction form for the systematic review of advances in analytical approaches for background parenchymal enhancement in predicting breast tumor response to neoadjuvant chemotherapy. (DOCX) [file pone.0317240.s004.docx]

**S3: Data extraction form for the systematic review of advances in analytical approaches for background parenchymal enhancement in predicting breast tumor response to neoadjuvant chemotherapy**

|  | Research Review Questions | Response |
| --- | --- | --- |
| **Study Attributes** | | |
| 1. | First Author | ---------------------- |
| 2. | Year of Publication | ---------------------- |
| 3. | Title of Publication | ---------------------- |
| 4. | Study Design | ---------------------- |
| **Study Population** | | |
| 5. | Sample Size | ---------------------- |
| 6. | Mean Age | ---------------------- |
| **BPE Analysis Techniques** | | |
| 7. | BPE Analysis Approach | (1) Single pre-and-post BPE Analysis  (2) Longitudinal Analysis |
| 8. | Number of DCE-MRI examinations during NAC | 🞏 Baseline-T0  🞏 Early Treatment-T1  🞏 Inter-regimen-T2  🞏 Pre-Surgery-T3 |
| 9. | Methods for region of interest (ROI) segmentation | (1) Manual  (2) Semi-automated  (3) Fully Automated (Others)  (4) Fully Automated (Deep learning) |
| 10. | BPE assessment methods | (1) Qualitative  (2) Quantitative  (3) Both |
| 11. | BPE quantification methods | ----------------------------- |
| **Tumor Attributes** | | |
| 12. | Tumor subtype | ----------------------------- |
| 13. | Phase when response was first detected after NAC | (1) Early Treatment Phase  (2) Inter-regimen Phase  (3) Post NAC, before Surgery Phase |
| **Nature of Data Components** | | |
| 14. | Type of data analyzed to predict response to NAC | (1) Clinicopathologic  (2) Qualitative/Quantified BPE  (3) Both |
| **Techniques for model Development** | | |
| 15. | Statistical/Artificial Intelligence techniques to predict response | ---------------------------------- |
| **Study Limitations** | | |
| 16. | Any study limitations | ------------------------------------ |
| **Study recommendations** | | |
| 17. | Any future recommendations | -------------------------------------- |
