# Supplementary material for: Advances in analytical approaches for background parenchymal enhancement in predicting breast tumor response to neoadjuvant chemotherapy: A systematic review
Source: PLoS One. 2025 Mar 7;20(3):e0317240. doi: 10.1371/journal.pone.0317240 (PMC11888135; doi:10.1371/journal.pone.0317240)
Supplement: S1 Table — The overall risk of bias assessment and applicability concerns for the selected studies derived using the revised Quality Assessment of Diagnostic Accuracy Studies (QUADAS-2) tool. (DOCX) [file pone.0317240.s008.docx]

**Table 1. Risk of bias assessment and applicability concerns**

| **Study Reference** | **RISK OF BIAS** | | | | **APPLICABILITY CONCERNS** | | |
| --- | --- | --- | --- | --- | --- | --- | --- |
|  | Patient Selection | Index Test | Reference Standard | Flow And Timing | Patient Selection | Index Test | Reference Standard |
| Preibsch et al., 2016 [54] | Low | Low | Low | Unclear | Low | Low | Low |
| Chen et al., 2015 [36] | Low | Unclear | Low | High | Low | Low | Low |
| You et al., 2018 [55] | Low | Low | Low | Unclear | Low | Low | Low |
| La Forgia et al., 2021 [28] | Low | Unclear | Low | Low | Low | Low | Low |
| Seon Jeong Oh et al., 2018 [56] | Low | Unclear | Low | Unclear | Low | Low | Low |
| Dong et al., 2018 [32] | Low | Low | Low | Unclear | Low | Low | Low |
| You et al., 2017 [50] | Low | Unclear | Low | Low | Low | Low | Low |
| Arasu et al., 2020 [51] | Low | Unclear | Low | Unclear | Low | Low | Low |
| Xin Huang et al., 2023 [57] | Low | Low | Low | Low | Low | Low | Low |
| Nguyen et al., 2020 [58] | Low | Low | Low | Low | Low | Low | Low |
| R. Rella et al., 2020 [59] | Low | Unclear | Low | Unclear | Low | Low | Low |
| Li et al., 2020 [60] | Low | Low | Low | Low | Low | Low | Low |
| Onishi et al., 2021 [53] | Low | Unclear | Low | Unclear | Low | Low | Low |

The overall risk of bias assessment and applicability concerns for the selected studies derived using the revised Quality Assessment of Diagnostic Accuracy Studies (QUADAS-2) tool.
