# Supplementary material for: Advances in analytical approaches for background parenchymal enhancement in predicting breast tumor response to neoadjuvant chemotherapy: A systematic review
Source: PLoS One. 2025 Mar 7;20(3):e0317240. doi: 10.1371/journal.pone.0317240 (PMC11888135; doi:10.1371/journal.pone.0317240)
Supplement: S2 Table — Overview of the studies that evaluated relationship between BPE and NAC response. (DOCX) [file pone.0317240.s009.docx]

**Table 2. Relationship between BPE and NAC response**

| **STUDY** | | | **STUDY POPULATION** | | **DATA** | |
| --- | --- | --- | --- | --- | --- | --- |
| Study Reference | Year | Study Design | Sample Size | Subtype | Type | # Excluded for missing one or more MRI timepoints |
| Preibsch et al., 2016 [54] | 2016 | Retrospective, single center | 73 | Invasive ductal, invasive lobular or other carcinomas | BPE | 8 |
| Chen et al., 2015 [36] | 2015 | Retrospective, single center | 46 | Invasive ductal cancer, infiltrating lobular cancer | BPE | NR |
| You et al., 2018 [55] | 2018 | Retrospective, single center | 71 | Unilateral human epidermal growth factor receptor 2 (HER2) positive breast cancer | BPE | NR |
| La Forgia et al., 2021 [28] | 2021 | Retrospective, single center | 80 | Stage I to stage II (Luminal A, Luminal B, HER2 positive, Triple Negative, Triple Positive) | BPE; FGT | NR |
| Oh et al., 2018 [56] | 2018 | Retrospective, Single center | 186 | Invasive breast cancer | BPE | 75 |
| Dong et al., 2018 [32] | 2018 | Retrospective, single center | 51 | HER2-positive breast cancer | BPE | 3 |
| You et al., 2017 [50] | 2017 | Retrospective, single center | 90 | Unilateral invasive ductal carcinomas, ductal carcinoma in suit, invasive lobular carcinoma | BPE; breast tumor size | 11 |
| Arasu et al., 2020 [51] | 2020 | Prospective, multiple center | 88 | HR+HER2− advanced breast cancer (stage II or III) | BPE; FTV | 1 |
| Xin Huang et al., 2023 [57] | 2023 | Retrospective, multicenter study | 894 | Invasive breast cancer | BPE | 242 |
| Nguyen et al., 2020 [58] | 2020 | Retrospective, multicenter study | 990 | Stage II/III breast cancer (HR and HER2) | BPE | 255 |
| Rella et al., 2020 [59] | 2020 | Retrospective, single center | 228 | Unilateral invasive breast cancer | BPE | 10 |
| Li et al., 2020 [60] | 2020 | Retrospective, multiple center | 384 | Stage II or III unilateral breast cancer (HER2 and HR) | BPE, FTV, LD, and SPH | 96 |
| Onishi et al., 2021 [53] | 2021 | Retrospective, multicenter | 882 | Unilateral breast cancer (hormone receptor (HR)-positive and HR-negative) | BPE | 88 |

Overview of the studies that evaluated relationship between BPE and NAC response.

Abbreviations: # = Number; BPE= background parenchymal enhancement; FGT = Fibroglandular Tissue; FTV= Functional Tumor Volume; LD = Longest diameter; Sphericity =SPH; NR= Not Reported
