# Supplementary material for: Advances in analytical approaches for background parenchymal enhancement in predicting breast tumor response to neoadjuvant chemotherapy: A systematic review
Source: PLoS One. 2025 Mar 7;20(3):e0317240. doi: 10.1371/journal.pone.0317240 (PMC11888135; doi:10.1371/journal.pone.0317240)
Supplement: S3 Table — Overview of the analytical methods utilized for BPE in predicting breast tumor response to neoadjuvant chemotherapy. (DOCX) [file pone.0317240.s010.docx]

**Table 3. BPE analytical methods**

| Study Reference | BPE Analysis Approach | Methods for BPE Change Analysis | BPE-DCE-MRI Examinations Time points | ROI Segmentation method | BPE Assessment Methods | BPE Quantification Methods | Phase when Significant Association Between change in BPE and pCR First Detected | Methods to determine whether change in BPE can predict Tumor-Response |
| --- | --- | --- | --- | --- | --- | --- | --- | --- |
| Preibsch et al., 2016 [54] | Single time-point BPE Analysis | Comparison of qualitative changes in baseline BPE versus post-NAC BPE | Before Initiation of NAC (baseline), after NAC completion | NA | Qualitative | NA | Late phase of NAC treatment | Correlation Analysis |
| Chen et al., 2015 [36] | Longitudinal (Multiple time-points) Analysis | %ΔBPE at Early phase, Mid-Time point phase compared to the %ΔBPE at Baseline | Before Initiation of NAC (baseline), Early phase (2 to 4 weeks), NAC mid-time point (after 8 weeks) | Computer-based segmentation algorithm | Quantitative | The average of the contrast enhancements measured from all pixels contained with the segmented fibroglandular tissue | Early Phase of NAC | Two-tailed t test |
| You et al., 2018 [55] | Single time-point BPE Analysis | Comparison of qualitative changes in baseline BPE versus BPE after 2nd NAC cycle | Before Initiation of NAC (baseline), after 2nd NAC cycle | NA | Qualitative | NA | 2nd NAC cycle | Statistical methods |
| La Forgia et al., 2021 [28] | Longitudinal (Multiple time-points) Analysis | Comparison of qualitative changes in baseline BPE versus post-NAC BPEs (BPE-Intermediate step, BPE-Final step) | Before Initiation of NAC (baseline), after at least 3 months of treatment (step 1/intermediate step or the middle step/ after 4 rounds of therapy), after NAC completion (step II or the final step) | NA | Qualitative | NA | Early Phase of NAC | Statistical tests (Chi-square test) |
| Seon Jeong Oh et al., 2018 [56] | Single time-point BPE Analysis | Comparison of qualitative changes in baseline BPE versus post-NAC BPEs | Before Initiation of NAC (baseline), post-NAC BPE | NA | Qualitative | NA | Late phase of NAC treatment | Analysis of variance |
| Dong et al., 2018 [32] | Single time-point BPE Analysis | Comparison of qualitative changes in baseline BPE versus post-NAC BPE | Before Initiation of NAC (baseline), after NAC completion | NA | Qualitative | NA | Late phase of NAC treatment | Wilcoxon test |
| You et al., 2017 [50] | Longitudinal (Multiple time-points) Analysis | %ΔBPE1/2/3 = (BPE2nd/4th/6th follow-up MRI – BPE baseline MRI)/ BPE baseline MRI *100% | Before initiation of NAC (baseline), and after 2nd, 4th, 6th and 8th NAC | Fully automated scheme | Quantitative | The enhanced fibroglandular tissue volume/total fibroglandular tissue volume) × 100% | Early Treatment (T1) | Binary Logistic regression |
| Arasu et al., 2020 [51] | Longitudinal (Multiple time-points) Analysis | %ΔBPE_1/2/3 = (BPE_1/2/3 – BPE_0 MRI)/ BPE_0 MRI *100% | Before initiation of NAC (baseline (T0)), after 3 weeks of therapy (early treatment (T1)), after 12 weeks of therapy (inter-regimen (T2)), and after NAC completion and prior to surgery (pre-surgery (T3)) | Whole breast manual segmentation | Quantitative | Average of early enhancement measured for all voxel of segmented fibroglandular tissue | Early Treatment (T1) | Logistic regression models |
| Xin Huang et al., 2023 [57] | Longitudinal (Multiple time-points) Analysis | %ΔBPET1/T2 = (BPE T1/T2 – BPE T0 MRI)/ BPE T0 MRI *100% | Pre-treatment (T0), early treatment (T1), and inter-regimen (T2) | Deep learning-based segmentation method (nnU-Net) | Quantitative | Averaging the percent enhancement values for all voxels in the masked volume | Early treatment (T1) phase | Univariate logistic regression |
| Nguyen et al., 2020 [58] | Longitudinal (Multiple time-points) Analysis | %ΔBPET1/T2/T3= (BPE T1/T2/T3 – BPE T0 MRI)/ BPE T0 MRI *100% | Baseline (T0), after 3weeks of treatment (T1), after 12weeks of treatment and between drug regimens (T2), and after completion of treatment (T3) | Fully automated segmentation methods (full and half stack segmentation methods) | Quantitative | Averaging the percent enhancement values for all voxels in the masked volume | Early treatment (T1) phase | Univariate logistic regression |
| Rella et al., 2020 [59] | Longitudinal (Multiple time-points) Analysis | %ΔBPET1/T2/T3= (BPE T1/T2/T3 – BPE T0 MRI)/ BPE T0 MRI *100% | Before (within 4 weeks before NAC), at mid-point (after the 4^th^ NAC cycle, before next cycle), and after (within 2 weeks after NAC, prior to surgery) NAC | Semi-automated breast segmentation | Quantitative | The enhancement rate identified in the MRI after chemotherapy minus the enhancement rate identified at the baseline MRI (total BPE change). | Early treatment phase | Fisher Exact test |
| Li et al., 2020 [60] | Longitudinal (Multiple time-points) Analysis | %ΔBPE at T1, T2, T3 compared to the %ΔBPE at Baseline (T0) | Pre-treatment (T0), after 3 cycles (T1, early NAC), after 12 cycles and between drug regimens (T2, mid-NAC), and before surgery (T3, post-NAC) | Fully Automated algorithm | Quantitative | Mean percent enhancement of fibroglandular tissue in the contralateral breast | NR | Logistic regression |
| Onishi et al., 2021 [53] | Longitudinal (Multiple time-points) Analysis | %ΔBPE at T1, T2, T3 compared to the %ΔBPE at Baseline (T0) | Before treatment (T0), early treatment (T1), interregimen (T2), and before surgery (T3) | Automated fibroglandular tissue segmentation | Quantitative | By averaging the percent enhancement values for all voxels in the masked volume | HR–positive (Interregimen, T2)  HR–negative (Early treatment, T1) | The Fisher exact test |

Overview of the analytical methods utilized for BPE in predicting breast tumor response to neoadjuvant chemotherapy.

Abbreviations: BPE= background parenchymal enhancement; NAC = Neoadjuvant Chemotherapy; NR= Not Reported; NA= not applicable
