# Supplementary figures and images for: Advances in analytical approaches for background parenchymal enhancement in predicting breast tumor response to neoadjuvant chemotherapy: A systematic review
Source: PLoS One. 2025 Mar 7;20(3):e0317240. doi: 10.1371/journal.pone.0317240 (PMC11888135; doi:10.1371/journal.pone.0317240)

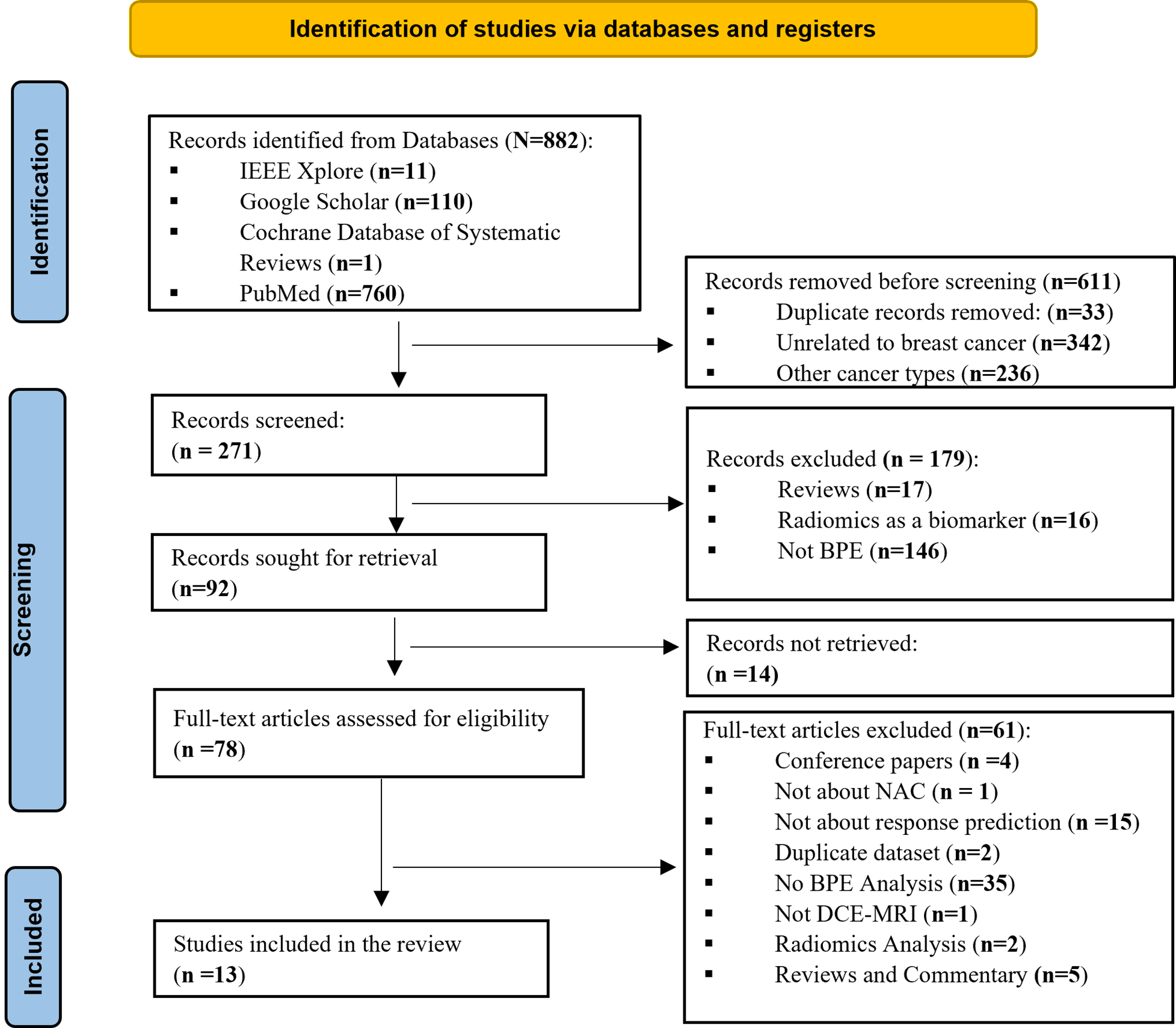

Supplement: S1 Fig — Flow diagram of preferred reporting items for systematic reviews and meta-analyses (PRISMA). (TIF) [file pone.0317240.s011.tif]

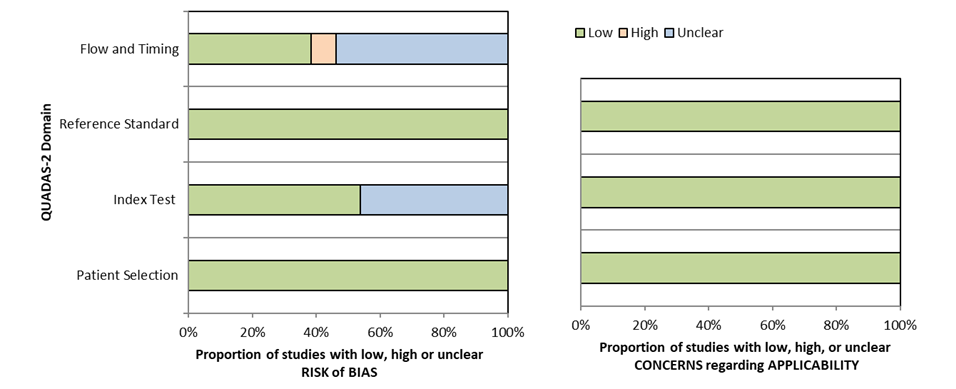

Supplement: S2 Fig — Grouped bar graph of risk of bias and applicability concerns for the selected studies derived using the revised Quality Assessment of Diagnostic Accuracy Studies (QUADAS-2) tool. (TIF) [file pone.0317240.s012.tif]
