# Supplementary material for: Advances in analytical approaches for background parenchymal enhancement in predicting breast tumor response to neoadjuvant chemotherapy: A systematic review
Source: PLoS One. 2025 Mar 7;20(3):e0317240. doi: 10.1371/journal.pone.0317240 (PMC11888135; doi:10.1371/journal.pone.0317240)
Supplement: Attachment — Submitted filename: Response to Reviewers.docx [file pone.0317240.s013.docx]

**Rebuttal letter**

**For the manuscript “**Advances in analytical approaches for background parenchymal enhancement in predicting breast tumor response to neoadjuvant chemotherapy: A comprehensive review**” [PONE-D-24-30545]**

9^th^ December, 2024

**Response to reviewers**

We sincerely thank the editor and reviewers for their valuable time and thoughtful evaluation of our manuscript. To address the feedback provided, we have crafted detailed, line-by-line responses to each comment. For clarity, our responses are highlighted in blue and correspond directly to the comments or questions, which are marked in yellow. Additionally, we have referenced the review decision from the editorial manager and maintained the same format to structure our responses systematically.

**INITIAL COMMENTS**

PONE-D-24-30545

Advances in analytical approaches for background parenchymal enhancement in predicting breast tumor response to neoadjuvant chemotherapy: A comprehensive review

PLOS ONE

Dear Dr. Thomas,

Thank you for submitting your manuscript to PLOS ONE. After careful consideration, we feel that it has merit but does not fully meet PLOS ONE’s publication criteria as it currently stands. Therefore, we invite you to submit a revised version of the manuscript that addresses the points raised during the review process.


Please submit your revised manuscript by Dec 13 2024 11:59PM. If you will need more time than this to complete your revisions, please reply to this message or contact the journal office at [plosone@plos.org](mailto:plosone@plos.org). When you're ready to submit your revision, log on to <https://www.editorialmanager.com/pone/> and select the 'Submissions Needing Revision' folder to locate your manuscript file.

Please include the following items when submitting your revised manuscript:

- A rebuttal letter that responds to each point raised by the academic editor and reviewer(s). You should upload this letter as a separate file labeled 'Response to Reviewers'.
- A marked-up copy of your manuscript that highlights changes made to the original version. You should upload this as a separate file labeled 'Revised Manuscript with Track Changes'.
- An unmarked version of your revised paper without tracked changes. You should upload this as a separate file labeled 'Manuscript'.

If you would like to make changes to your financial disclosure, please include your updated statement in your cover letter. Guidelines for resubmitting your figure files are available below the reviewer comments at the end of this letter.

If applicable, we recommend that you deposit your laboratory protocols in [protocols.io](http://protocols.io/) to enhance the reproducibility of your results. Protocols.io assigns your protocol its own identifier (DOI) so that it can be cited independently in the future. For instructions see: [https://journals.plos.org/plosone/s/submission-guidelines#loc-laboratory-protocols](https://track.editorialmanager.com/CL0/https:%2F%2Fjournals.plos.org%2Fplosone%2Fs%2Fsubmission-guidelines%23loc-laboratory-protocols/1/010f0192da375242-ab905f0d-5e14-4c13-be48-79b74237d3ef-000000/cNd1LlWr7Miued8tmbtsEx04QLTRARbi3IsBV5cISQ4=183). Additionally, PLOS ONE offers an option for publishing peer-reviewed Lab Protocol articles, which describe protocols hosted on [protocols.io](http://protocols.io/). Read more information on sharing protocols at [https://plos.org/protocols?utm_medium=editorial-email&utm_source=authorletters&utm_campaign=protocols](https://track.editorialmanager.com/CL0/https:%2F%2Fplos.org%2Fprotocols%3Futm_medium=editorial-email%26utm_source=authorletters%26utm_campaign=protocols/1/010f0192da375242-ab905f0d-5e14-4c13-be48-79b74237d3ef-000000/rU9vSoI2O8ep4zHQV_-qwHVsq86Yl6LCzRZCHZAQWIc=183).

We look forward to receiving your revised manuscript.

Response: Thank you for the constructive feedback and helpful guidance on protocol registration and reproducibility resources. We are pleased to confirm that we have already registered and published our protocol with the International Prospective Register of Systematic Reviews (Protocol Identifier: CRD42024574312) on September 8, 2024 (<http://www.crd.york.ac.uk/PROSPERO/>).

For your review, we have incorporated this identifier into the manuscript (please refer to Materials and Methods section, page 6).

Kind regards,

Jiang Gui
Academic Editor
PLOS ONE

Journal Requirements:

When submitting your revision, we need you to address these additional requirements.

1. Please ensure that your manuscript meets PLOS ONE's style requirements, including those for file naming. The PLOS ONE style templates can be found at 
<https://journals.plos.org/plosone/s/file?id=wjVg/PLOSOne_formatting_sample_main_body.pdf> and 
<https://journals.plos.org/plosone/s/file?id=ba62/PLOSOne_formatting_sample_title_authors_affiliations.pdf>

Response: Thank you for pointing this out. We have carefully reviewed the PLOS ONE style requirements and ensured that our manuscript fully complies, including file naming conventions, bibliography styles, abstract, introduction, and figure formatting. We greatly appreciated the provided style templates, which were invaluable in guiding our revisions.

2. Thank you for stating the following financial disclosure: 
 Deutscher Akademischer Austauschdienst scholarship (DAAD) under the 2020/2023 In-Country/In-Region Scholarship Programme in Eastern Africa -Kenya (grant 91601895).

Please state what role the funders took in the study.  If the funders had no role, please state: ""The funders had no role in study design, data collection and analysis, decision to publish, or preparation of the manuscript."" 
If this statement is not correct you must amend it as needed.

Please respond by return e-mail so that we can amend your financial disclosure and competing interests on your behalf.

Response: We commend the guidance regarding the financial disclosure! We confirm that the statement is correct as written: "The funders had no role in study design, data collection and analysis, decision to publish, or preparation of the manuscript". Thank you for your assistance in finalizing these details.

3. Please review your reference list to ensure that it is complete and correct. If you have cited papers that have been retracted, please include the rationale for doing so in the manuscript text, or remove these references and replace them with relevant current references. Any changes to the reference list should be mentioned in the rebuttal letter that accompanies your revised manuscript. If you need to cite a retracted article, indicate the article’s retracted status in the References list and also include a citation and full reference for the retraction notice.

Response: Thank you for this important reminder. We have carefully reviewed our reference list to confirm its completeness and accuracy. We verified that none of the cited papers have been retracted; however, we have only updated the two references to Numbered style for uniformity.

Additional Editor Comments:

Dear Authors,

Please the address the minor concerns raised by reviewer 1 regarding on introduction and reference sections. Thanks.


[Note: HTML markup is below. Please do not edit.]

Reviewers' comments:

Reviewer's Responses to Questions

**Comments to the Author**

1. Is the manuscript technically sound, and do the data support the conclusions?

The manuscript must describe a technically sound piece of scientific research with data that supports the conclusions. Experiments must have been conducted rigorously, with appropriate controls, replication, and sample sizes. The conclusions must be drawn appropriately based on the data presented.

Reviewer #1: Yes

2. Has the statistical analysis been performed appropriately and rigorously?

Reviewer #1: Yes

3. Have the authors made all data underlying the findings in their manuscript fully available?

The [PLOS Data policy](http://track.editorialmanager.com/CL0/http:%2F%2Fwww.plosone.org%2Fstatic%2Fpolicies.action%23sharing/1/010f0192da375242-ab905f0d-5e14-4c13-be48-79b74237d3ef-000000/GbwO9Px-x53PFXyOxafwgp9JHa7xJ48-E4D4hBkgASo=183) requires authors to make all data underlying the findings described in their manuscript fully available without restriction, with rare exception (please refer to the Data Availability Statement in the manuscript PDF file). The data should be provided as part of the manuscript or its supporting information, or deposited to a public repository. For example, in addition to summary statistics, the data points behind means, medians and variance measures should be available. If there are restrictions on publicly sharing data—e.g., participant privacy or use of data from a third party—those must be specified.

Reviewer #1: Yes

4. Is the manuscript presented in an intelligible fashion and written in standard English?

PLOS ONE does not copyedit accepted manuscripts, so the language in submitted articles must be clear, correct, and unambiguous. Any typographical or grammatical errors should be corrected at revision, so please note any specific errors here.

Reviewer #1: Yes

5. Review Comments to the Author

Please use the space provided to explain your answers to the questions above. You may also include additional comments for the author, including concerns about dual publication, research ethics, or publication ethics. (Please upload your review as an attachment if it exceeds 20,000 characters)

Reviewer #1: This systematic review is important as it synthesizes current knowledge on BPE as a biomarker for predicting breast tumor response to NAC, addressing both advancements and challenges in the field. It contrasts traditional single time-point analyses with longitudinal approaches, emphasizing their advantages in capturing dynamic changes. The review emphasizes the significance of personalized treatment strategies and the integration of various data types, ultimately aiming to enhance patient outcomes through improved predictive models in breast cancer management.

The introduction is well-structured and informative.

The methodology section is robust, comprehensive and well-aligned with PLOS ONE's rigorous standards. Multiple databases (PubMed, Google Scholar, IEEE Xplore, Cochrane) were used and search strategy with Boolean operators according to PICO framework enhances the precision and clarity. The risk of bias was rigorously evaluated by including modified QUADAS-2 tool. The PRISMA checklist, search terms according to PICO framework, and inclusion/exclusion criteria are well-documented, contributing to transparency and reproducibility.

The discussion identifies limitations in current methodologies, including subjective assessments and challenges with missing data. Recommendations for standardizing BPE measurement and incorporating personalized patient factors are made to enhance predictive accuracy. Overall, the review provides valuable insights into the complexities of utilizing BPE in clinical settings, highlighting the need for a multimodal approach in treatment strategies.

Just a few minor suggestions before to proceed publishing:

Comment:
• While the introduction is logical and cites relevant literature, it would benefit from a more explicit discussion of gaps in the existing research that your review aims to address. (i.e. more on BPE change analysis.) This would strengthen the justification and comprehension of the study.

Response: Thank you for your constructive feedback to enhance the clarity and relevance of our work. We appreciate your suggestion and agree that an expanded discussion of the existing gaps in BPE change analysis could better highlight the unique contributions of our review. We have revised the manuscript’s introduction to provide a more detailed analysis of these gaps, specifically focusing on the current limitations in longitudinal BPE change analysis (please refer to Introduction section, page 5 and 6).

Comment:
• Two of the references were not provided as numbers, should be corrected.

Response: Thank you for your careful review. We have now updated the two references to Numbered style and ensured accurate numbering in the revised manuscript version (please see Introduction section, page 4).

6. PLOS authors have the option to publish the peer review history of their article ([what does this mean?](https://track.editorialmanager.com/CL0/https:%2F%2Fjournals.plos.org%2Fplosone%2Fs%2Feditorial-and-peer-review-process%23loc-peer-review-history/1/010f0192da375242-ab905f0d-5e14-4c13-be48-79b74237d3ef-000000/GbYzXOW6UnAMs2XlE18yw7YghfeSFyStWyW85JBWYSQ=183)). If published, this will include your full peer review and any attached files.


If you choose “no”, your identity will remain anonymous but your review may still be made public.


**Do you want your identity to be public for this peer review?** For information about this choice, including consent withdrawal, please see our [Privacy Policy](https://track.editorialmanager.com/CL0/https:%2F%2Fwww.plos.org%2Fprivacy-policy/1/010f0192da375242-ab905f0d-5e14-4c13-be48-79b74237d3ef-000000/8ZY5PVkoQa7pU2CacMeON7a2UmLYmBbd3UTG7mROugA=183).

Reviewer #1: No

[NOTE: If reviewer comments were submitted as an attachment file, they will be attached to this email and accessible via the submission site. Please log into your account, locate the manuscript record, and check for the action link "View Attachments". If this link does not appear, there are no attachment files.]

Comment:
While revising your submission, please upload your figure files to the Preflight Analysis and Conversion Engine (PACE) digital diagnostic tool, [https://pacev2.apexcovantage.com/](https://track.editorialmanager.com/CL0/https:%2F%2Fpacev2.apexcovantage.com%2F/1/010f0192da375242-ab905f0d-5e14-4c13-be48-79b74237d3ef-000000/TY99JLoQp9p3ZRRr_wMBa0c4dU6FerV_NKVqamT1eWc=183). PACE helps ensure that figures meet PLOS requirements. To use PACE, you must first register as a user. Registration is free. Then, login and navigate to the UPLOAD tab, where you will find detailed instructions on how to use the tool. If you encounter any issues or have any questions when using PACE, please email PLOS at [figures@plos.org](mailto:figures@plos.org). Please note that Supporting Information files do not need this step.

Response: We have registered with PACE, and all figures are consistent with PLOS guidelines. We downloaded them from PACE and uploaded them as Fig 1 and Fig 2 figures in TIFF format.

**ADDITIONAL COMMENTS**

PONE-D-24-30545R1
Advances in analytical approaches for background parenchymal enhancement in predicting breast tumor response to neoadjuvant chemotherapy: A comprehensive review

Dear Dr. Thomas,

We've checked your submission and before we can proceed, we need you to address the following issues:

Comment:
1. We note that your Data Availability Statement is currently as follows:

"All relevant data are within the manuscript and its Supporting Information files."

Please confirm at this time whether or not your submission contains all raw data required to replicate the results of your study. Authors must share the “minimal data set” for their submission. PLOS defines the minimal data set to consist of the data required to replicate all study findings reported in the article, as well as related metadata and methods (<https://journals.plos.org/plosone/s/data-availability#loc-minimal-data-set-definition>).

For example, authors should submit the following data:
- The values behind the means, standard deviations and other measures reported;
- The values used to build graphs;
- The points extracted from images for analysis.

Authors do not need to submit their entire data set if only a portion of the data was used in the reported study.

If your submission does not contain these data, please either upload them as Supporting Information files or deposit them to a stable, public repository and provide us with the relevant URLs, DOIs, or accession numbers. For a list of recommended repositories, please see <https://journals.plos.org/plosone/s/recommended-repositories>.

If there are ethical or legal restrictions on sharing a de-identified data set, please explain them in detail (e.g., data contain potentially sensitive information, data are owned by a third-party organization, etc.) and who has imposed them (e.g., an ethics committee). Please also provide contact information for a data access committee, ethics committee, or other institutional body to which data requests may be sent. If data are owned by a third party, please indicate how others may request data access.

Response: Thank you for bringing this up. We confirm that the minimal dataset required to replicate the results of our study has been uploaded as part of the Supporting Information files accompanying the manuscript. This dataset includes:

- Table of Studies Identified in the Literature Search (refer to ‘Table of all Studies’);
- Table of Extracted Data from the primary research sources for the systematic review (refer to ‘Table of Extracted Data’);
- Table of Risk of Bias and applicability concerns for the selected studies based on revised Quality Assessment of Diagnostic Accuracy Studies (QUADAS-2) tool (refer to ‘Table of Risk of Bias and Quality Assessment’);
- A table of values used to build grouped bar graph of risk of bias and applicability concerns for the selected studies derived using the revised Quality Assessment of Diagnostic Accuracy Studies (QUADAS-2) tool (refer to ‘Table of Grouped Bar Graph of Risk of Bias and Applicability Concerns’);

All relevant data and supporting materials are available within the manuscript and its Supporting Information files, ensuring transparency and compliance with PLOS' data availability requirements. Should additional clarification or details be required, we are happy to provide further information.

Thank you for your attention to this matter.

Comment:
2. Please remove your figures/ from within your manuscript file, leaving only the individual TIFF/EPS image files. These will be automatically included in the reviewer’s PDF.

Response: Thank you for your helpful comment regarding the placement of figures in the manuscript. We have removed the figures from the main manuscript file and ensured that all individual TIFF/EPS image files and tables are uploaded separately. We appreciate your guidance and remain committed to following the journal's submission guidelines.

Comment:
3. Thank you for submitting your revised manuscript. As required by our policy on Data Availability, could you also please ensure your manuscript or supplementary information includes the following information.

Please accept our apologies for not sending you this request sooner.

1) A numbered table of all studies identified in the literature search, including those that were excluded from the analyses.

a) For every excluded study, the table should list the reason(s) for exclusion.

b) If any of the included studies are unpublished, include a link (URL) to the primary source or detailed information about how the content can be accessed.

2) A table of all data extracted from the primary research sources for the systematic review and/or meta-analysis. The table must include the following information for each study:

a) Name of data extractors and date of data extraction

b) Confirmation that the study was eligible to be included in the review.

c) All data extracted from each study for the reported systematic review and/or meta-analysis that would be needed to replicate your analyses.

If data or supporting information were obtained from another source (e.g. correspondence with the author of the original research article), please provide the source of data and dates on which the data/information were obtained by your research group.

3) If applicable for your analysis, a table showing the completed risk of bias and quality/certainty assessments for each study or outcome. Please ensure this is provided for each domain or parameter assessed. For example, if you used the Cochrane risk-of-bias tool for randomized trials, provide answers to each of the signaling questions for each study. If you used GRADE to assess certainty of evidence, provide judgements about each of the quality of evidence factor. This should be provided for each outcome.

4) An explanation of how missing data were handled.

This information can be included in the main text, supplementary information, or relevant data repository. Please note that providing these underlying data is a requirement for publication in this journal, and if these data are not provided your manuscript might be rejected.

Response: Thank you for your thorough review and for providing these detailed requirements for ensuring compliance with the Data Availability policy. We appreciate your diligence and the opportunity to address these points.

We confirm that the manuscript and/or supplementary information include all the requested elements as follows:

1. **Numbered Table of Studies Identified in the Literature Search**:
   - A complete table of all studies identified during the literature search is included (Please refer to ‘Table of all Studies’)
   - For excluded studies, the table provides the reasons for exclusion.
   - No unpublished studies were included, hence no links to the primary sources or detailed access information are provided.
2. **Table of Extracted Data**:
   - A comprehensive table containing all data extracted from the primary research sources for the systematic review is included (refer to ‘Table of Extracted Data’).
   - This table details:
     a) The names of data extractors and the dates of data extraction.
     b) Confirmation of each study’s eligibility for inclusion.
     c) All extracted data necessary to replicate the analyses.
3. **Risk of Bias and Quality/Certainty Assessments**:
   - A table presenting the completed risk of bias and quality for each study and outcome is included (refer to ‘Table of Risk of Bias and Quality Assessment’).
4. **Explanation of Handling Missing Data**:
   - A detailed explanation of the methods used to handle missing data is included in the manuscript.

We hope this addresses your concerns fully. Please let us know if there are any additional adjustments or clarifications required.

Thank you once again for your valuable feedback and guidance.

Comment:
In addition to the above, please note that a simple title correction is required: The title should include the term "Systematic Review" (as required by item 1 of the PRISMA checklist). Please ensure this is updated in the manuscript file and the online submission information.

Response: Thank you for your comment. The title has been updated to include the term "Systematic Review" in accordance with item 1 of the PRISMA checklist. This correction has been applied to both the manuscript file and the online submission information. Please let us know if further adjustments are needed.

We've returned your manuscript to your account. Please resolve these issues and resubmit your manuscript within 21 days. If you need more time, please email the journal office at [plosone@plos.org](mailto:plosone@plos.org). We are happy to grant extensions of up to one month past this due date. If we do not hear from you within 21 days, we will withdraw your manuscript.

Please log on to PLOS Editorial Manager at <https://www.editorialmanager.com/pone/> to access your manuscript. You will find your manuscript in the 'Submissions Sent Back to Author' link under the New Submissions menu. Be sure to remove your previous manuscript file if you are uploading a new file in response to these requests. After you've made the changes requested above, please be sure to view and approve the revised PDF after rebuilding the PDF to complete the resubmission process.

We are requesting these changes to comply with the PLOS ONE submission guidelines (<https://journals.plos.org/plosone/s/submission-guidelines>). Please note that we won't send your manuscript for review until you have resolved the above requests.

Thank you for submitting your work to PLOS ONE and supporting our mission of Open Science.


Kind regards,

Oriel Jerome Delas Alas Vida
PLOS ONE
